# Preoperative Prediction of Microvascular Invasion in Hepatocellular Carcinoma *via* Multi-Parametric MRI Radiomics

**DOI:** 10.3389/fonc.2021.633596

**Published:** 2021-03-03

**Authors:** Yang Zhang, Zhenyu Shu, Qin Ye, Junfa Chen, Jianguo Zhong, Hongyang Jiang, Cuiyun Wu, Taihen Yu, Peipei Pang, Tianshi Ma, Chunmiao Lin

**Affiliations:** ^1^Department of Radiology, Zhejiang Provincial People’s Hospital, People’s Hospital of Hangzhou Medical College, Hangzhou, China; ^2^Department of Pharmaceuticals Diagnosis, GE Healthcare, Hangzhou, China; ^3^Department of Pathology, Zhejiang Provincial People’s Hospital, People’s Hospital of Hangzhou Medical College, Hangzhou, China

**Keywords:** hepatocellular carcinoma, microvascular invasion, multi-parametric MRI, radiomics, models

## Abstract

**Objectives:**

To systematically evaluate and compare the predictive capability for microvascular invasion (MVI) in hepatocellular carcinoma (HCC) patients based on radiomics from multi-parametric MRI (mp-MRI) including six sequences when used individually or combined, and to establish and validate the optimal combined model.

**Methods:**

A total of 195 patients confirmed HCC were divided into training (n = 136) and validation (n = 59) datasets. All volumes of interest of tumors were respectively segmented on T_2_-weighted imaging, diffusion-weighted imaging, apparent diffusion coefficient, artery phase, portal venous phase, and delay phase sequences, from which quantitative radiomics features were extracted and analyzed individually or combined. Multivariate logistic regression analyses were undertaken to construct clinical model, respective single-sequence radiomics models, fusion radiomics models based on different sequences and combined model. The accuracy, sensitivity, specificity and area under the receiver operating characteristic curve (AUC) were calculated to evaluate the performance of different models.

**Results:**

Among nine radiomics models, the model from all sequences performed best with AUCs 0.889 and 0.822 in the training and validation datasets, respectively. The combined model incorporating radiomics from all sequences and effective clinical features achieved satisfactory preoperative prediction of MVI with AUCs 0.901 and 0.840, respectively, and could identify the higher risk population of MVI (P < 0.001). The Delong test manifested significant differences with P < 0.001 in the training dataset and P = 0.005 in the validation dataset between the combined model and clinical model.

**Conclusions:**

The combined model can preoperatively and noninvasively predict MVI in HCC patients and may act as a usefully clinical tool to guide subsequent individualized treatment.

## Introduction

Hepatocellular carcinoma (HCC) accounts for 75%–85% of primary liver cancer, which is the sixth most common cancer and the fourth leading cause of cancer-related death globally (1, 2). Hepatectomy and liver transplantation are potential effective treatments for HCC. However, the prognosis is still poor with high tumor recurrence rate of 70% after hepatectomy and 25% after transplantation (3, 4). Microvascular invasion (MVI) is regarded as an extremely important independent risk factor of postoperative recurrence and poor outcome (5, 6). Study found that patients with higher MVI risk benefited from anatomical hepatectomy and widened surgical margin in terms of disease-free survival and overall survival (7). Unfortunately, the diagnosis of MVI still depends on the postoperative histopathology in current clinical practice (8). Therefore, early and accurate preoperative prediction of the MVI is of vital importance for clinical decision-making in choosing the best strategy to manage the individual HCC patients (9).

Currently, many efforts have been made to preoperatively find the factors related to MVI, including clinical biomarkers and imaging features. According to previous researches (10–12), serum total bilirubin (TBil), hepatitis B surface antigen (HBsAg), neutrophils to lymphocytes ratio (NLR), alpha-fetoprotein (AFP), and tumor maximum diameter (MD) were related to MVI. However, significant biomarkers varied from study to study. Serum AFP lacked sensitivity for the prediction of MVI (13). Besides, some biomarkers, such as TBil and NLR, also fluctuated under the influence of time, eating, exercise, and so on. Various researches based on preoperative imaging features of HCC were performed to predict the MVI, but showed no consensus (14–16). Moreover, the reproducibility and practicality were still controversial due to the overreliance on the subjective judgment of radiologists (17). All of these lack characteristic evaluation on tumor heterogeneity that reflects different biological behaviors of HCC.

Radiomics, as an emerging method for medical image processing, is used to convert medical images into high-throughput quantification features that greatly push the development of precision medicine (18). In contrast to tissue biomarkers, radiomics noninvasively reflect the entire tumor at the heterogeneity, phenotype and microenvironment (19). Recently, a few studies have been published on the utility of radiomics for MVI prediction in HCC patients based on CT (20–22) and hepatobiliary-phase MRI (23, 24). They found that radiomics was more desirable than conventional and functional imaging methods, and the results appeared to be preliminary but encouraging (25). Multi-parametric MRI (mp-MRI) includes different sequences, indicating its greater potential to assess tumor metabolism and proliferation with higher accuracy (26).

Therefore, in this study, we aimed to systematically evaluate and compare the predictive capability for MVI in HCC patients based on radiomics from mp-MRI including six sequences when used individually or combined, and to establish and validate the optimal combined model.

## Materials and Methods

### Patients

**Figure 1:**
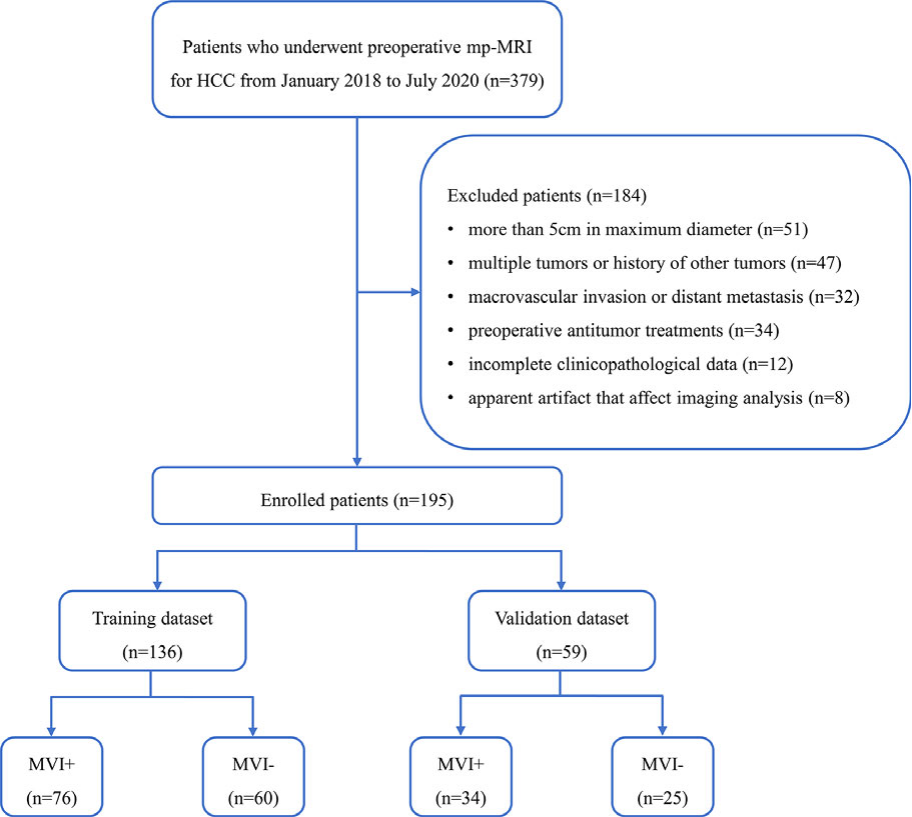
Flowchart of the enrolled patients. (HCC, hepatocellular carcinoma; mp-MRI, multi-parametric MRI; MVI+, patients with microvascular invasion; MVI−, patients without microvascular invasion).

This retrospective study was approved by our institutional ethics committee and informed consent was waived. Between January 2018 and July 2020, 379 patients underwent preoperative mp-MRI for HCC in our institutional database. All data were acquired from the picture archiving and communication system (PACS), which was used to identify patients who had pathologically confirmed MVI-positive (MVI+) or MVI-negative (MVI−). Inclusion criteria included: ①pathologically confirmed HCC; ②single tumor with maximum diameter no more than 5 cm; ③no macrovascular invasion and no distant metastasis; ④underwent radical resection; ⑤no cancer-related treatments before mp-MRI and operation; and⑥mp-MRI with sufficient image quality. A total of 184 patients were excluded because of the following reasons: ①more than 5 cm in maximum diameter (n = 51); ② multiple tumors or history of other tumors (n = 47); ③ macrovascular invasion or distant metastasis (n = 32); ④preoperative antitumor treatments (n =3 4); ⑤incomplete clinicopathological data (n = 12); or ⑥ apparent artifact that affect imaging analysis (n = 8). Finally, 195 patients were enrolled and were divided into training (n = 136) and validation (n = 59) datasets at a ratio of 7:3 according to the time of mp-MRI examination. The patient recruitment process is shown in **Figure 1**.

### Clinical Data

The preoperative clinical data were collected from our PACS, including age, gender (male or female), HBsAg status (positive or negative), NLR level, AFP level, TBil level, MD, and tumor location (left or right lobe).

### MRI Protocol

All patients received mp-MRI examinations one week before surgery using a 3.0T MRI scanner (Discovery MR 750, GE Healthcare, Waukesha, WI, United States). Our liver mp-MRI protocol included axial T_2_-weighted imaging (T_2_WI) with fat suppression, dual-echo (in-phase and opposed-phase) T_1_-weighted imaging (T_1_WI), diffusion-weighted imaging (DWI), apparent diffusion coefficient (ADC), pre-contrast and post-contrast dynamic three-dimensional fast-spoiled gradient-recalled echo sequence (liver acceleration volume acquisition, LAVA). DWI was obtained using a respiratory triggering, a single-shot echo-planar imaging pulse sequence with b values of 0 and 800 s/mm^2^. Dynamic contrast-enhanced (DCE) LAVA images were acquired at 15–20s (arterial phase, AP), 50–55s (portal venous phase, PP) and 85–90s (delayed phase, DP) after contrast-agent injection. The scanning parameters were described as **Table 1**.

**Table 1:** Imaging protocol parameters for multi-parametric MRI.

Sequences	TR (msec)	TE (msec)	FOV (mm²)	matrix	thickness (mm)	interslice gap (mm)	NEX
T_2_WI	13,000	75	360 × 360	320 × 320	5	1	1.5
T_1_WI	3.7	1.7	360 × 288	260 × 224	5	0	1.0
DWI	8,000	50	360 × 288	128 × 96	5	1	2.0
DCE	3.7	1.7	360 × 288	260 × 224	5	0	1.0

TR, repetition time; TE, echo time; FOV, field of view; NEX, number of excitations; DCE, dynamic contrast-enhanced.

### Image Segmentation and Feature Selection

Image preprocessing including resample, intensity normalization and gray-level discretization were performed with AK software (Artificial Intelligence Kit V3.0.0.R, GE Healthcare) as described previously (27). Then, we got the standardized images with 1 × 1 × 1 mm^3^ voxel size for each sequence. Three-dimensional manual segmentation of the entire HCC was performed by an abdominal radiologist with more than 10 years of experience in liver MRI using the ITK-SNAP software (28). All volumes of interest (VOIs) were respectively segmented on the standardized T_2_WI, DWI with b values of 800 s/mm^2^, ADC, AP, PP, DP sequences slice-by-slice for each patient. The segmentation results were then corrected and validated by a senior radiologist with more than 15 years of experience in liver MRI. To ensure reproducibility and repeatability, the inter-reader reliability on VOI segmentation was computed by comparing the measurement from these two experienced radiologists in a cohort of 30 randomly selected patients by Spearman’s rank correlation test. The two radiologists were completely blinded to the clinical, laboratory, and pathologic information.

All VOIs were imported into the AK software for feature extraction, including histogram, gray-level cooccurrence matrix (GLCM), gray-level size zone matrix (GLSZM), run-length matrix (RLM), formfactor, and haralick. A total of 396 radiomics features were extracted for each patient each sequence, including histogram (42 features), GLCM (144 features), GLSZM (11 features), RLM (180 features), formfactor (9 features), and haralick (10 features). The details of all radiomics features are described in **Supplementary Figure S1**. Firstly, Spearman’s rank correlation test was performed on a cohort of 30 randomly selected patients not only to test the repeatability, but also to exclude the radiomics features with correlation coefficients lower than 0.80. Then, dimension reduction was performed using analysis of variance and Mann-Whitney U-test, correlation analysis and the least absolute shrinkage and selection operator (LASSO) to reduce data redundancy and to further select significant radiomics features. The details of radiomics features extraction and selection for each sequence are described in Supplementary Radiomics Feature.

### Model Construction and Evaluation

In order to preoperatively predict MVI, multivariate logistic regression analysis was undertaken to construct a total of 11 models, including one clinical model, nine radiomics models and one combined model. The clinical model was constructed by integrating the final selected clinical features using logistic regression modeling. The radiomics models were built using the respective remaining features. And radiomics models included six single-sequence models based on T_2_WI, DWI, ADC, AP, PP, and DP, respectively, and three fusion models based on AP, PP and DP sequences (DCE), T_2_WI and DP sequences (T_2_WI&DP), all sequences (ALL), respectively. The combined model incorporated radiomics from all sequences and effective clinical features together with a logistic regression model.

Thereafter, we calculated the radiomics score for each patient. The predictive efficiency of different models in both the training and validation datasets was then evaluated using the area under the receiver operating characteristic (ROC) curve (AUC). Calibration curve was used to assess the calibration. Further, decision curve analysis (DCA) was conducted to assess the clinical efficiency of different models in predicting MVI.

### Statistical Analysis

Statistical analyses were performed with R software (version 3.4.1) and SPSS software (version 24.0). Continuous variables were expressed as mean ± standard deviation or median (interquartile range). Categorical variables were presented as numbers (percentages). The normality of distribution was evaluated using the Kolmogorov-Smirnov test. The two-sample t test, Mann-Whitney U test, and Chi-square test were used to identify variables differed significantly between the training and validation datasets. Then, LASSO logistic regression model with penalty parameter tuning was conducted by 10-fold cross-validation based on the minimum criteria to select the most valuable predictive features. Forward stepwise selection was applied through a likelihood ratio test and Akaike’s information criterion as the stopping rule. Multivariate logistic regression analysis was used to construct prediction models. ROC curves were used to evaluate the predictive efficiency of different models in both the training and validation datasets. The ‘‘rms’’ package of R software was used to construct calibration plots. The “dca.R.” package of R software was used to construct DCA. Statistical significance was set at P < 0.05.

## Results

### Patients’ Characteristics

In this study, a total of 195 patients were included with an average age of 57.65 ± 10.80 years (range: 27–83 years; median: 56.00 years), and were divided into training (n = 136) and validation (n = 59) datasets. Patients’ characteristics in the training and validation datasets were fully detailed in **Table 2**. There were no statistical differences between the training and validation datasets (P = 0.139–0.722). We observed statistical differences between the MVI+ and MVI− groups in terms of age (P = 0.044), AFP (P = 0.001), MD (P = 0.014) in the training dataset and AFP (P = 0.003) in the validation dataset. There were no significant differences between the MVI+ and MVI− groups in terms of gender, HBsAg, NLR, TBil, and tumor location both in the training and in the validation datasets.

**Table 2:** Patients’ characteristics in the training and validation datasets.

Characteristics	Training dataset (n = 136)			Validation dataset (n = 59)			P_inter_
	MVI+ (n = 76)	MVI− (n = 60)	P_intra_	MVI+ (n = 34)	MVI− (n = 25)	P_intra_	
Age (years), median (IQR)	55.50 (51.00–64.00)	60.00 (53.00–68.75)	0.044^*^	54.50 (49.00–66.25)	54.00 (51.50–68.00)	0.480	0.459
Gender, no. (%)							
Male	65 (85.5)	56 (93.3)	0.243	30 (88.2)	20 (80.0)	0.615	0.409
Female	11 (14.5)	4 (6.7)		4 (11.8)	5 (20.0)		
HBsAg, no. (%)							
Positive	61 (80.3)	44 (73.3)	0.339	31 (91.2)	20 (80.0)	0.393	0.139
Negative	15 (19.7)	16 (26.7)		3 (8.8)	5 (20.0)		
NLR, median (IQR)	3.10 (1.84–5.53)	2.45 (1.51–4.98)	0.329	2.56 (1.76–4.24)	1.95 (1.31–3.49)	0.163	0.150
AFP, median (IQR)	89.05 (6.60–787.38)	6.50 (3.23–89.40)	0.001^*^	159.60 (8.90–4,529.50)	6.30 (2.95–15.50)	0.003^*^	0.722
TBil, median (IQR)	15.05 (13.30–23.10)	18.45 (13.13–23.70)	0.302	16.50 (11.25–23.03)	15.60 (13.40–24.40)	0.505	0.695
MD (cm), median (IQR)	4.40 (3.05–5.00)	3.50 (2.05–4.68)	0.014^*^	4.00 (2.73–5.00)	3.20 (2.00–5.00)	0.214	0.650
Location, no. (%)							
Left lobe	24 (31.6)	24 (40.0)	0.308	12 (35.3)	10 (40.0)	0.712	0.294
Right lobe	52 (68.4)	36 (60.0)		22 (64.7)	15 (60.0)		

MVI+, patients with microvascular invasion; MVI-, patients without microvascular invasion; IQR, interquartile range; HBsAg, hepatitis B surface antigen; NLR, neutrophils to lymphocytes ratio; AFP, alpha-fetoprotein; TBil, serum total bilirubin; MD, tumor maximum diameter. P_intra_ is the result of univariate analyses between the MVI+ and MVI- groups while P_inter_ represents whether a significant difference exists between the training and validation datasets. ^*^represents P < 0.05.

### Models Construction

Univariate and multivariate analyses showed that age, AFP and MD were effective factors for clinical model construction.

**Figure 2:**
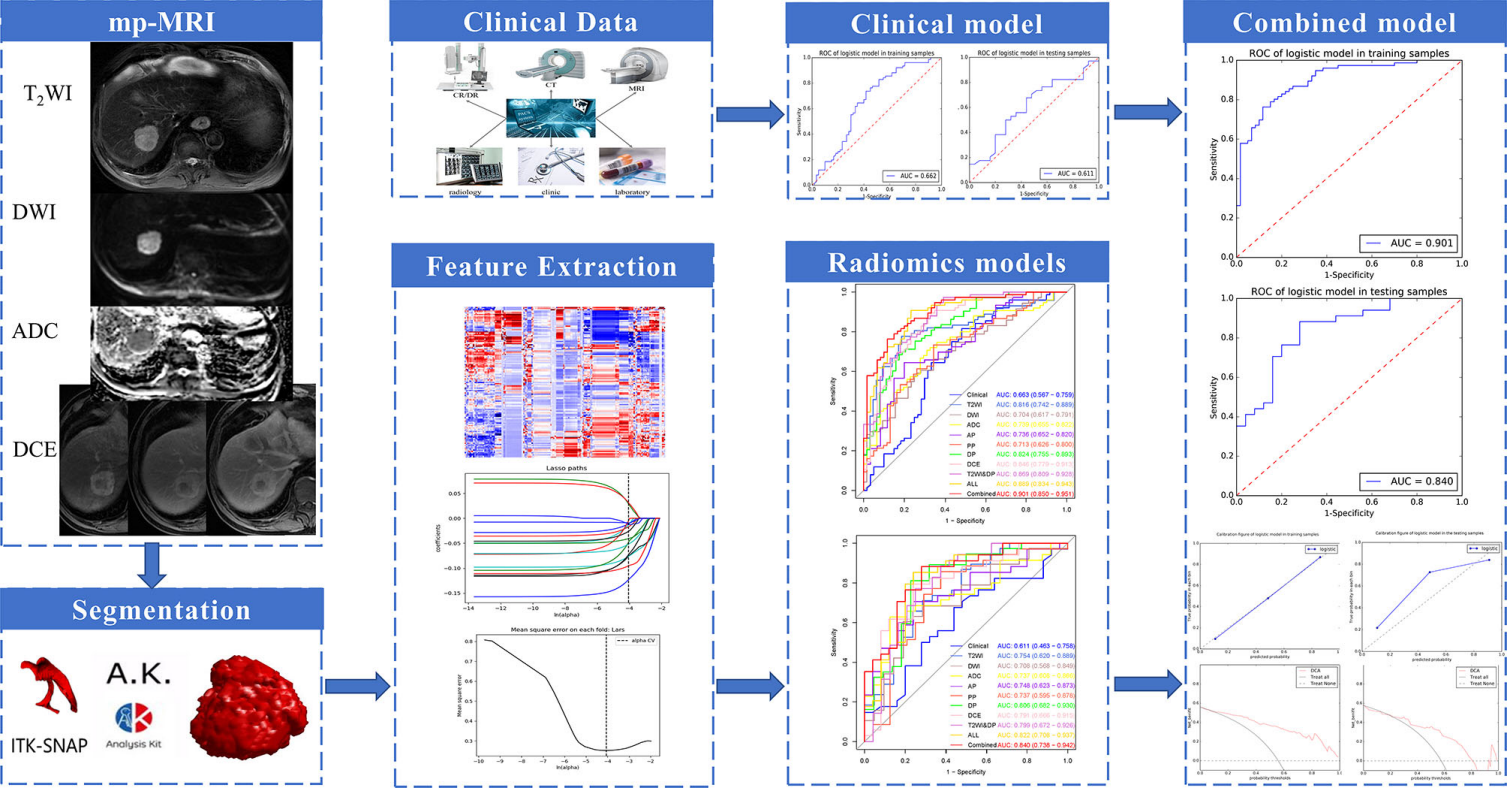
The radiomics workflow.

**Figure 2** showed the radiomics workflow. A total of 396 radiomics features were extracted for each patient each sequence. Spearman’s rank correlation test was performed to test the repeatability of radiomics features with correlation coefficients more than 0.80. Then, 378, 336, 351, 342, 327, 380 features were retained for T_2_WI, DWI, ADC, AP, PP, and DP, respectively. According to dimensionality reduction, 11, 7, 6, 8, 6, and 9 features were ultimately selected for T_2_WI, DWI, ADC, AP, PP, and DP radiomics model construction, respectively. Details of the selected radiomics features were shown in **Supplementary Table S1**.

### Performance of Different Models

**Figure 3:**
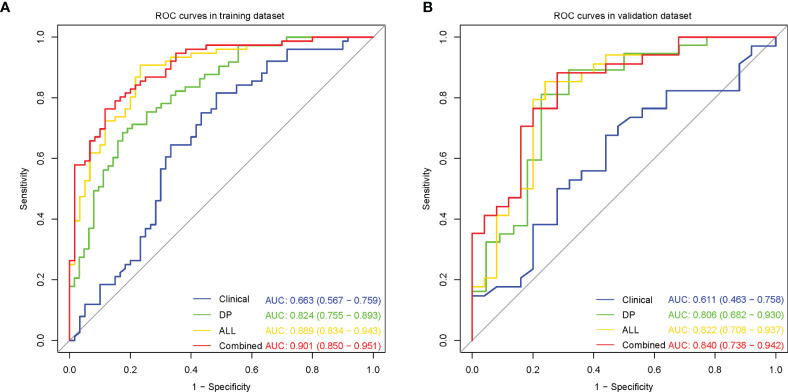
Receiver operating characteristic (ROC) curves for microvascular invasion prediction of different models in the training **(A)** and validation **(B)** datasets. (Clinical, the clinical model based on effective clinical parameters; DP, the DP radiomics model based on features from delayed phase; ALL, the fusion radiomics model based on features from all sequences; Combined, the combined model based on radiomics features from all sequences and effective clinical parameters).

The clinical model performed poorly with AUCs 0.663 and 0.611 in the training and validation datasets, respectively (**Figure 3**).

Among the six single-sequence radiomics models, we observed that the two best-performing models were T_2_WI (AUC = 0.816 and 0.754, respectively) and DP (AUC = 0.824 and 0.806, respectively), which outperformed the DWI (AUC = 0.704 and 0.708, respectively), ADC (AUC = 0.739 and 0.737, respectively), AP (AUC = 0.736 and 0.748, respectively), and PP (AUC = 0.713 and 0.737, respectively) models both in the training dataset and in the validation dataset (**Figure 3**, **Supplementary Figure S2A, B**).

Among the three fusion radiomics models, the ALL model had the best predictive performance with AUCs 0.889 and 0.822 in the training and validation datasets, respectively (**Figure 3**, **Supplementary Figures S2C, D**).

Then, incorporating radiomics from all sequences and effective clinical features to establish the optimal combined model, the predictive performance improved with AUCs 0.901 and 0.840 in the training and validation datasets, respectively (**Figure 3**). The AUC, sensitivity, specificity and accuracy for each model were summarized in **Table 3**.

**Table 3:** Predictive performance of different models.

Models	Training dataset (n = 136)				Validation dataset (n = 59)			
	AUC (95% CI)	Sensitivity	Specificity	Accuracy	AUC (95% CI)	Sensitivity	Specificity	Accuracy
Clinical	0.663 (0.567–0.759)	0.776	0.517	0.662	0.611 (0.463–0.758)	0.676	0.560	0.627
T_2_WI	0.816 (0.742–0.889)	0.819	0.656	0.743	0.754 (0.620–0.889)	0.737	0.667	0.712
DWI	0.704 (0.617–0.791)	0.732	0.547	0.644	0.708 (0.568–0.849)	0.684	0.571	0.644
ADC	0.739 (0.655–0.822)	0.720	0.672	0.699	0.737 (0.608–0.866)	0.743	0.500	0.644
AP	0.736 (0.652–0.820)	0.750	0.483	0.632	0.748 (0.623–0.873)	0.706	0.640	0.678
PP	0.713 (0.626–0.800)	0.827	0.410	0.640	0.737 (0.595–0.878)	0.857	0.542	0.729
DP	0.824 (0.755–0.893)	0.795	0.667	0.735	0.806 (0.682–0.930)	0.811	0.727	0.780
DCE	0.846 (0.779–0.913)	0.855	0.683	0.779	0.791 (0.666–0.915)	0.794	0.760	0.780
T_2_WI&DP	0.869 (0.809–0.928)	0.867	0.689	0.787	0.799 (0.672–0.926)	0.829	0.625	0.746
ALL	0.889 (0.834–0.943)	0.868	0.783	0.831	0.822 (0.708–0.937)	0.794	0.800	0.797
Combined	0.901 (0.850–0.951)	0.855	0.750	0.809	0.840 (0.738–0.942)	0.765	0.800	0.780

AP, arterial phase; PP, portal venous phase; DP, delay phase; DCE, dynamic contrast-enhanced; ALL, all sequences; Combined, incorporating radiomics from all sequences and effective clinical features; AUC, area under the curve; CI, confidence interval.

### Validation of Combined Model

**Figure 4:**
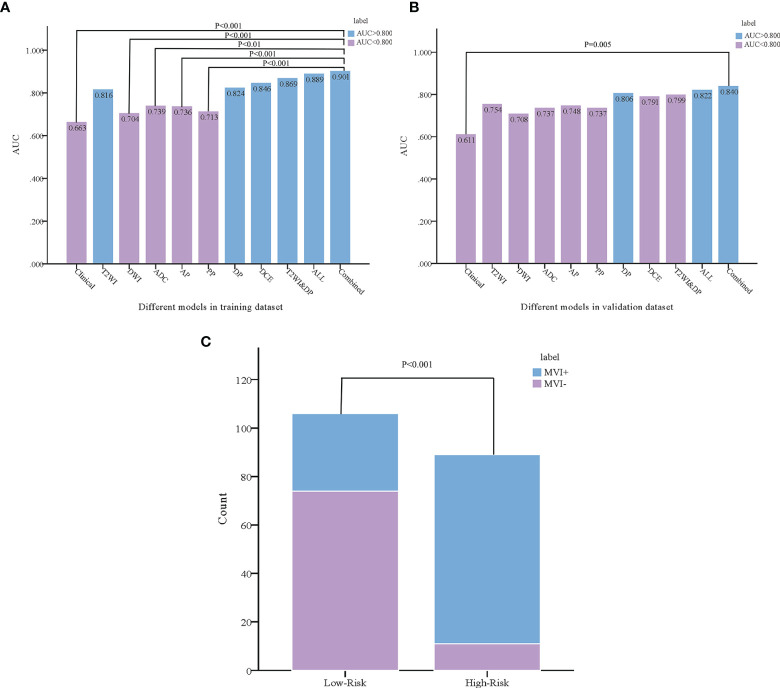
Comparison was made between the combined model and the other models using the Delong test in the training **(A)** and validation **(B)** datasets. According to the combined model, the probability of microvascular invasion in the high-risk group was significantly higher than that in the low-risk group **(C)**.

**Figure 5:**
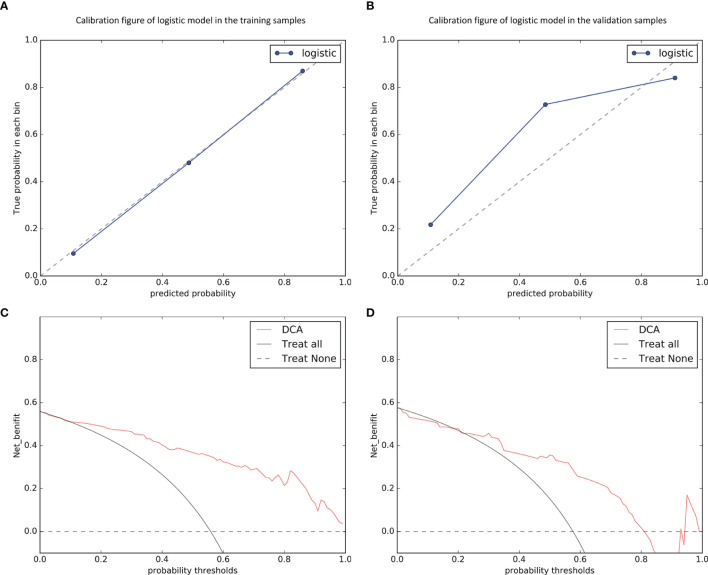
Calibration curves of the combined model in predicting microvascular invasion status on the training **(A)** and validation **(B)** datasets, which demonstrated good agreement with the ideal curve. The net benefit of the decision curve analysis (DCA) for the combined model was higher than the measures that treat all patients and treat none patient when the threshold probabilities were within 0.097–1.000 in the training dataset **(C)** and 0.219–0.806 in the validation dataset **(D)**.

As the combined model had the best predictive performance, we validated the efficacy of this model. Comparison was made between the combined model and the other models using the Delong test. It was worth mentioning that significant differences with P < 0.001 in the training dataset and P = 0.005 in the validation dataset between the combined model and the clinical model were achieved (**Figures 4A, B**). According to the optimal diagnostic cutoff value of 0.865, the patients were divided into high-risk group and low-risk group. There was a significant difference in predicting MVI status between the high-risk group and the low-risk group (P < 0.001), as shown in **Figure 4C**. Furthermore, the calibration curves demonstrated good consistency between the predicted and the actual MVI probability (**Figures 5A, B**). The Hosmer-Lemeshow test yielded no significant difference between the predictive calibration curve and the ideal curve for MVI prediction both in the training (P = 0.479) and in the validation datasets (P = 0.975), indicating no deviation from normality. DCA indicated the farther the decision curve was from the two extreme curves, the higher the clinical decision net benefit. In this study, the net benefit for the combined model was higher than the measures that treat all patients and treat none patient when the threshold probabilities were within 0.097–1.000 in the training dataset and 0.219–0.806 in the validation dataset (**Figures 5C, D**).

## Discussion

In this study, we systematically evaluated and compared the predictive capability for MVI in HCC patients based on radiomics from mp-MRI including six sequences when used individually or combined. It was noticed that the DP radiomics model (AUC = 0.824 and 0.806, respectively) outperformed other single-sequence models and the ALL radiomics model (AUC = 0.889 and 0.822, respectively) outperformed other fusion models both in training and in validation datasets. Furthermore, we established and validated an optimal combined model incorporating radiomics from all sequences and clinical features. Encouragingly, the results showed that the combined model can identify the higher risk population of MVI (P < 0.0001). This means that it is an accurate and effective tool that predicts MVI and may aid in clinical decision-making.

Recently, few studies about the utility of radiomics derived from mp-MRI in MVI preoperative prediction of HCC have been reported (29–31). Zhu et al. (29) only analyzed AP and PP radiomics and believed that AP radiomics model showed better diagnostic performance than did the PP model with AUC 0.773 vs. 0.623 in the validation dataset. Nebbia et al. (30) found the T_2_WI and PP radiomics models performed better with AUCs 0.808 and 0.792 to predict MVI. Another result (31) showed that first-order radiomics features extracted from PP (OR = 4.7, 95%CI 1.37–16.3, P = 0.014) were associated with MVI. However, they all ignored the preoperative prediction value for MVI based on radiomics derived from DP sequence. Besides, the last two studies were lack of further validation.

In this study, we studied six MRI sequences and systematically evaluated and compared their performance when used individually or combined. Interestingly, the predictive efficiency of each radiomics model was higher than that of clinical model indicating that the radiomics could act as a potential noninvasive biomarker to preoperatively predict MVI.

Among single-sequence radiomics models, the T_2_WI (AUC = 0.816 and 0.754, respectively) and the DP (AUC = 0.824 and 0.806, respectively) models outperformed other models both in training and in validation datasets. For T_2_WI, it was consistent with previous report with an AUC of 0.808 (30). However, that study only included 99 patients with unbalanced samples (61 MVI+ patients) and lacked further validation. In addition, only 14 cases with tumor size less than 3 cm in that study. For DP, this could be explained that the tumor continuously released large amounts of angiogenesis promoting factors, thus diversifying the neo-vasculature supplying blood of the tumor (32), changing the tumor perfusion, and leading to detectable differences in contrast-enhancement between MVI− and MVI+. The DP imaging are more valuable in showing the extravascular space and the vascular permeability. Therefore, DP is more valuable in predicting MVI in HCC patients. Previous study had reported that higher CT values in DP were closely related to MVI in HCC (33) and radiomics was a very promising non-invasive method for individualized evaluation based on intra-tumor heterogeneity analysis (34–36). These further confirmed our point.

Among fusion radiomics models, the ALL radiomics model performed best with AUCs 0.889 and 0.822 in the training and validation datasets, respectively. Previously, Zhang et al. (37) explored the radiomics model combining bi-regional radiomics features from DCE MRI, showing that the model can classify MVI+ and MVI– with AUCs 0.784 and 0.820 in the training and validation datasets, respectively, which was in accord with the results of our study. However, different from that study, our ALL radiomics model showed higher sensitivity both in the training (0.868 vs. 0.657) and in the validation (0.794 vs. 0.692) datasets. It was worth mentioning that Nebbia et al. (30) found that the performance of the model dropped when combining the tumor and margin radiomics features together, indicating that information from the margin was not complementary to (and may even be in conflict with) information from the tumor. Therefore, our study only used radiomics extracted from the whole tumors to predict MVI, and showed better predictive performance. In addition, our results revealed that different sequences can capture complementary information, thus achieving increased performance when combined. This is the first study to establish radiomics model from all MRI sequences to predict MVI in HCC so far.

Furthermore, we compared clinical model, different radiomics models and combined model in predicting MVI, which can provide references for subsequent treatments and selection of clinical evaluations. The results showed that the combined model achieved satisfactory preoperative prediction with good discrimination both in the training and validation datasets. Encouragingly, we found that the predictive ability of combined model (AUC = 0.901 and 0.840, respectively) significantly higher than that of clinical model (AUC = 0.663 and 0.611, respectively) both in the training dataset (P < 0.0001) and in the validation dataset (P = 0.0054). Moreover, the combined model showed better predictive performance than other radiomics models, although there were no statistically significant differences in the validation dataset. The sensitivity, specificity and accuracy of the combined model were more than 75%. More encouragingly, we found the combined model can identify the higher risk population of MVI. This means that it is an accurate and effective tool that predicts MVI and may aid in clinical decision-making. These findings showed that a multi-sequence approach to predict MVI was advisable.

From the clinical perspective, radiomics has the advantages of stable calculation, high repeatability, indefatigability, and free human subjective interference (38). The results of this study suggest that radiomics may be an option for preoperative prediction of MVI in HCC. This could alert pathologists to conduct more detailed pathological examination, especially when preoperative radiomics indicates a high likelihood of MVI. Meanwhile, predicting the possibility of MVI could also help clinicians to choose more suitable treatments for HCC patients.

The present study had the following advantages. First, different from previous researches, we studied six MRI sequences and systematically evaluated and compared their effects when used individually or combined. Second, the combined model we developed was based on routinely available radiologic images and clinical laboratory data and was easy to implement in routine clinical practice. Third, to the best of our knowledge, this was the first study incorporating radiomics from all sequences and clinical features to build a combined model for predicting the MVI status preoperatively and noninvasively. Last but not least, training and validation datasets were used to make the combined model more objective.

Despite our promising results, our study has some limitations. First, the sample size was limited to 195 patients. Nevertheless, we conducted an extensive analysis investigating six different MRI sequences. Second, this was a retrospective study using the same MRI scanner in a single-center. While this data consistency reduces potential confounding effects, external datasets and different MRI scanners are necessary to confirm the prediction value of the combined model. Third, because tumor VOIs were manually drawn, there could be some differences between the tumor VOI outlines in different sequences. More accurate and automatic tumor segmentation is needed for an in-depth analysis in future work.

In conclusion, our study suggests that radiomics based on mp-MRI can predict MVI in HCC preoperatively and noninvasively. The combined model incorporating radiomics from all sequences and clinical features can identify the higher risk population of MVI. It may be valuable for clinicians to objectively select appropriate treatments and as an individualized predictive tool for improving clinical outcomes.

## Data Availability Statement

The raw data supporting the conclusions of this article will be made available by the authors, without undue reservation.

## Ethics Statement

The studies involving human participants were reviewed and approved by Zhejiang Provincial People’s Hospital, People’s Hospital of Hangzhou Medical College. Written informed consent for participation was not required for this study in accordance with the national legislation and the institutional requirements.

## Author Contributions

CL proposed the study. YZ performed the research, analyzed the data, and wrote the first draft. ZS, QY, and PP auxiliarily analyzed the data. JC and JZ interpreted the data. HJ, CW, and TY collected the data. TM provided and reconfirmed all the pathological data. All authors contributed to the article and approved the submitted version.

## Funding

This study has received funding by the Medical Science and Technology Project of Zhejiang Province (No. 2021KY442), Zhejiang Traditional Chinese Medicine Administration (No. 2020ZA011), and Natural Science Foundation of Zhejiang Province (No. LGF21H180013).

## Conflict of Interest

PP was employed by GE Healthcare.

The authors declare that the research was conducted in the absence of any commercial or financial relationships that could be construed as a potential conflict of interest.
