# Supplementary material for: Preoperative Prediction of Microvascular Invasion in Hepatocellular Carcinoma via Multi-Parametric MRI Radiomics
Source: Front Oncol. 2021 Mar 3;11:633596. doi: 10.3389/fonc.2021.633596 (PMC7968223; doi:10.3389/fonc.2021.633596)
Supplement: Supplementary file 1 [file DataSheet_1.docx]

**Supplementary Material**

1. **Supplementary Methods**

**Supplementary Radiomics Features**

In this study, a total of 396 radiomics features were extracted using AK software (Artificial Intelligence Kit V3.0.0.R, GE Healthcare) for each patient each sequence, including standardized T_2_-weighted imaging (T_2_WI), diffusion-weighted imaging (DWI) with b values of 800 s/mm^2^, apparent diffusion coefficient (ADC), arterial phase (AP), portal venous phase (PP) and delayed phase (DP) sequences. Firstly, Spearman’s rank correlation test was performed on a cohort of 30 randomly selected patients not only to test the repeatability, but also to exclude the radiomics features with correlation coefficients lower than 0.80. Then, 378, 336, 351, 342, 327, 380 features were retained for T_2_WI, DWI, ADC, AP, PP and DP, respectively. Thereafter, we used analysis of variance, Mann-Whitney U-test, and correlation analysis to reduce data redundancy, and 28, 17, 15, 13, 14, 26 features were retained for T_2_WI, DWI, ADC, AP, PP and DP, respectively. Finally, the least absolute shrinkage and selection operator (LASSO) analysis was used to further select significant radiomics features, and 11, 7, 6, 8, 6 and 9 features were ultimately selected for T_2_WI, DWI, ADC, AP, PP and DP, respectively.

1. **Supplementary Figures and Table**

**Supplementary Figure S1.** The details of all radiomics features.

(Note. GLCM, gray-level cooccurrence matrix; GLSZM, gray-level size zone matrix; RLM, run-length matrix.)

**Supplementary Figure S2.** Receiver operating characteristic (ROC) curves for microvascular invasion (MVI) prediction of six single-sequence radiomics models in the training (A) and validation (B) datasets. ROC curves for MVI prediction of three fusion radiomics models in the training (C) and validation (D) datasets.

**Supplementary Table S1.** Selected radiomics features for the multi-parametric MRI

(Note. AP, arterial phase; PP, portal venous phase; DP, delay phase; GLCM, gray-level cooccurrence matrix; GLSZM, gray-level size zone matrix; RLM, run-length matrix.)
