# Supplementary figures and images for: Preoperative Prediction of Microvascular Invasion in Hepatocellular Carcinoma via Multi-Parametric MRI Radiomics
Source: Front Oncol. 2021 Mar 3;11:633596. doi: 10.3389/fonc.2021.633596 (PMC7968223; doi:10.3389/fonc.2021.633596)

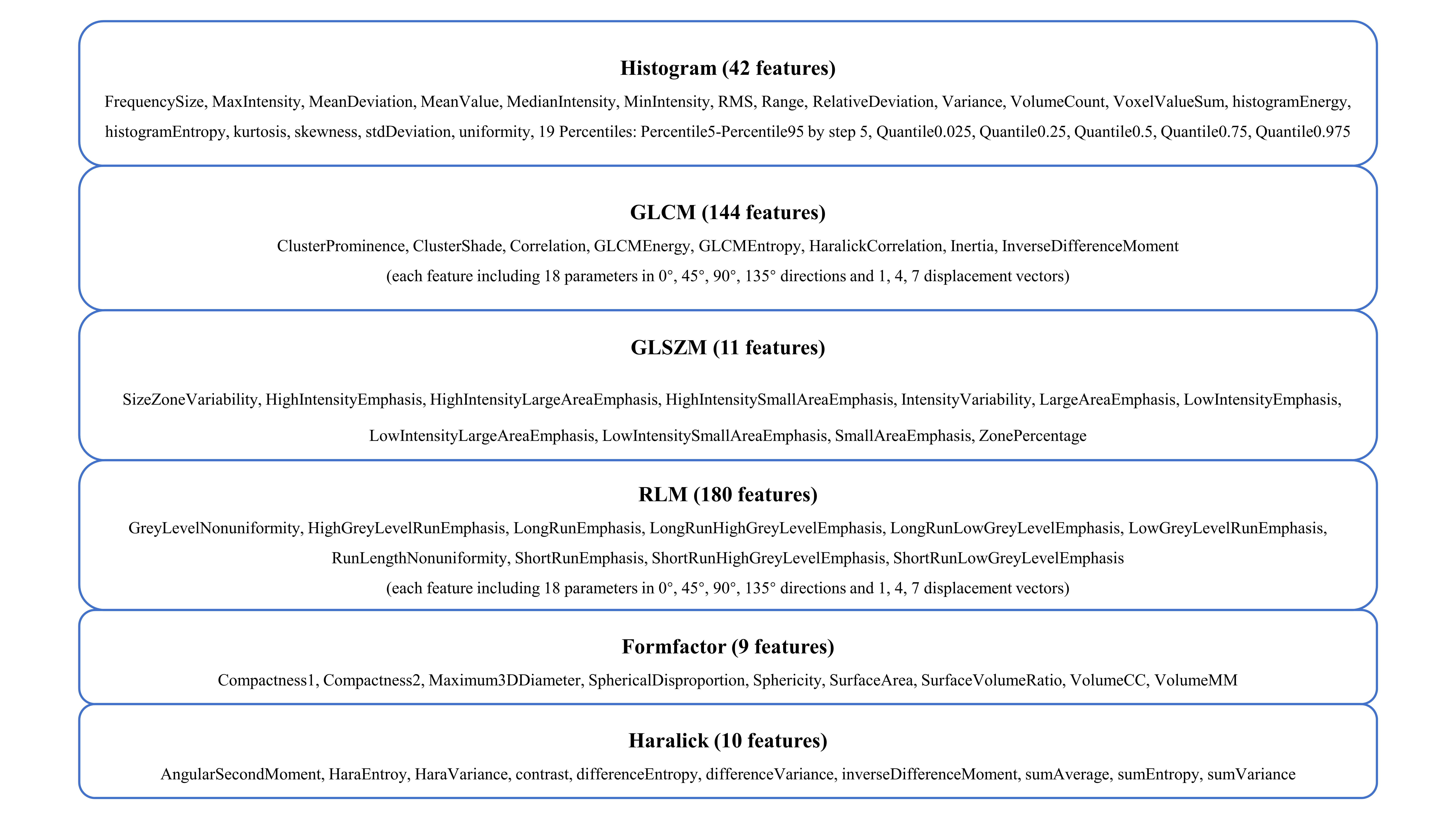

Supplement: Supplementary file 2 [file Image_1.tif]

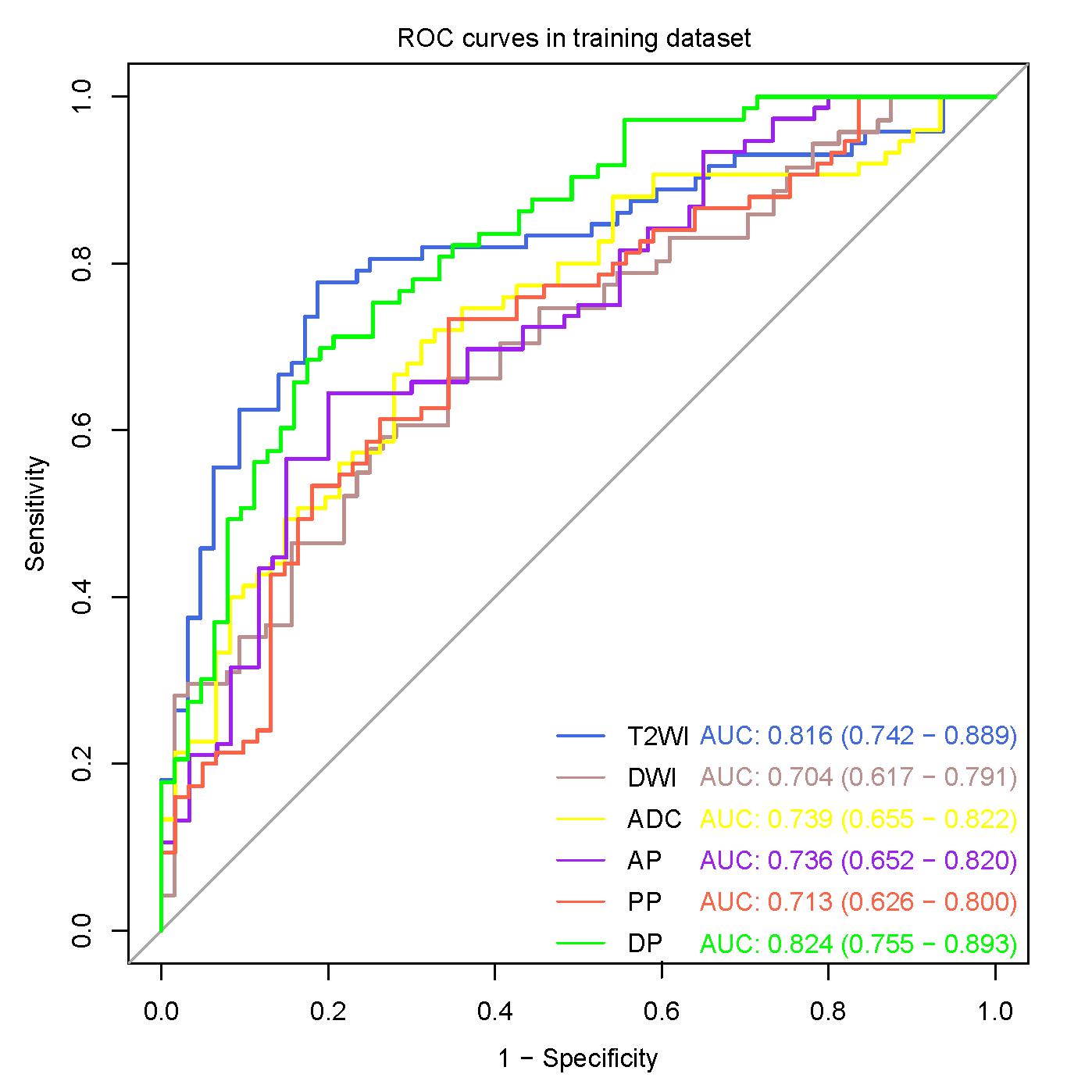

Supplement: Supplementary file 3 [file Image_2.tif]

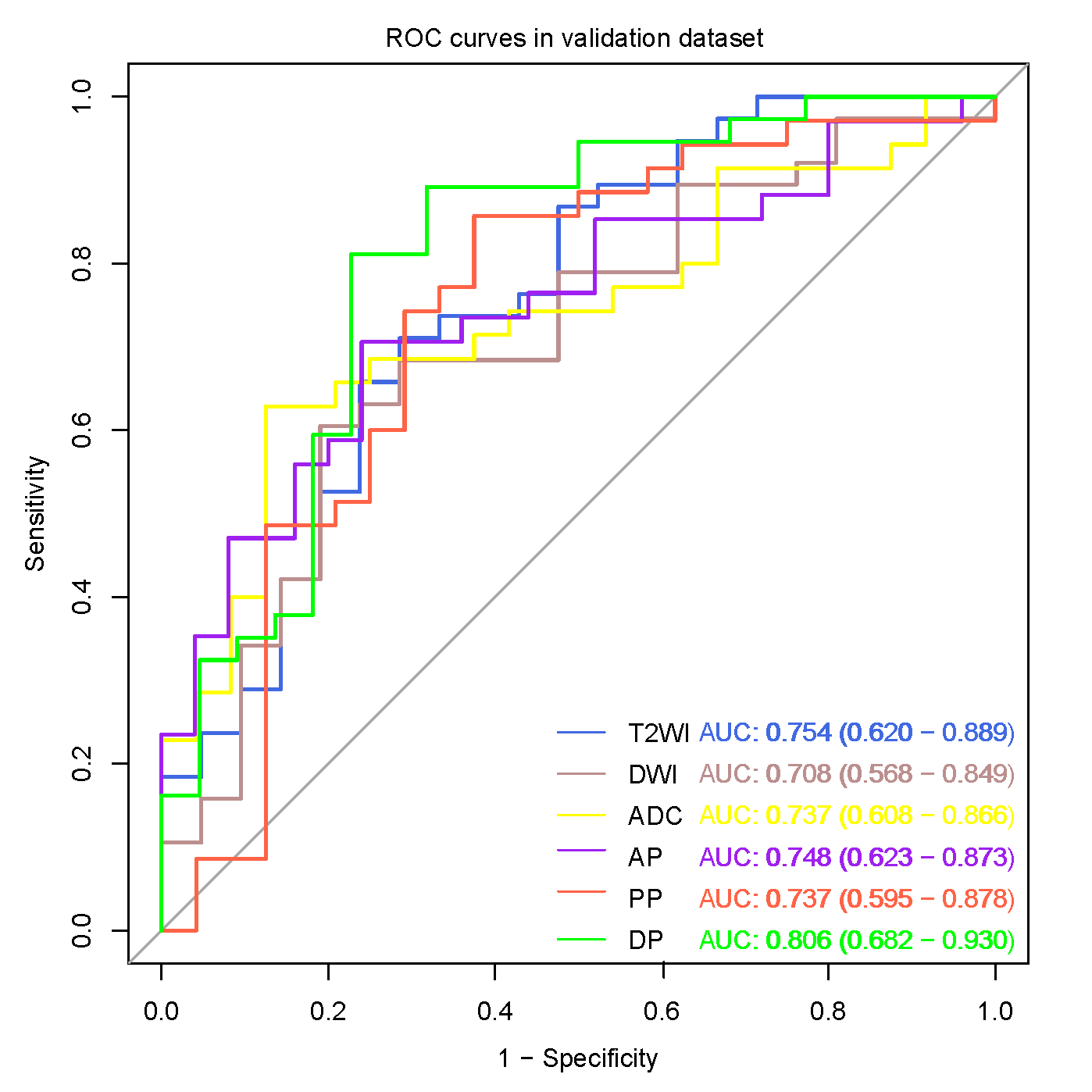

Supplement: Supplementary file 4 [file Image_3.tif]

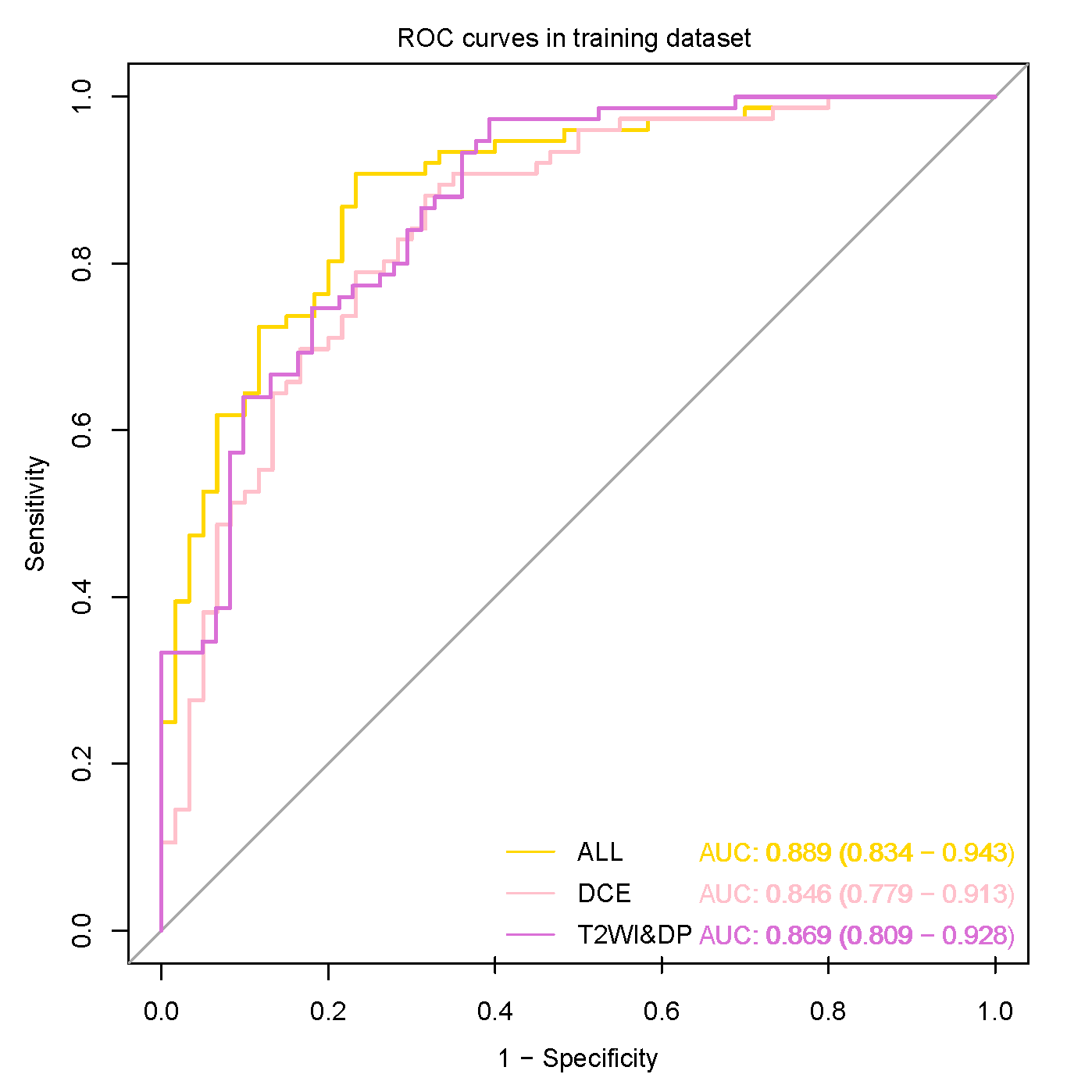

Supplement: Supplementary file 5 [file Image_4.tif]

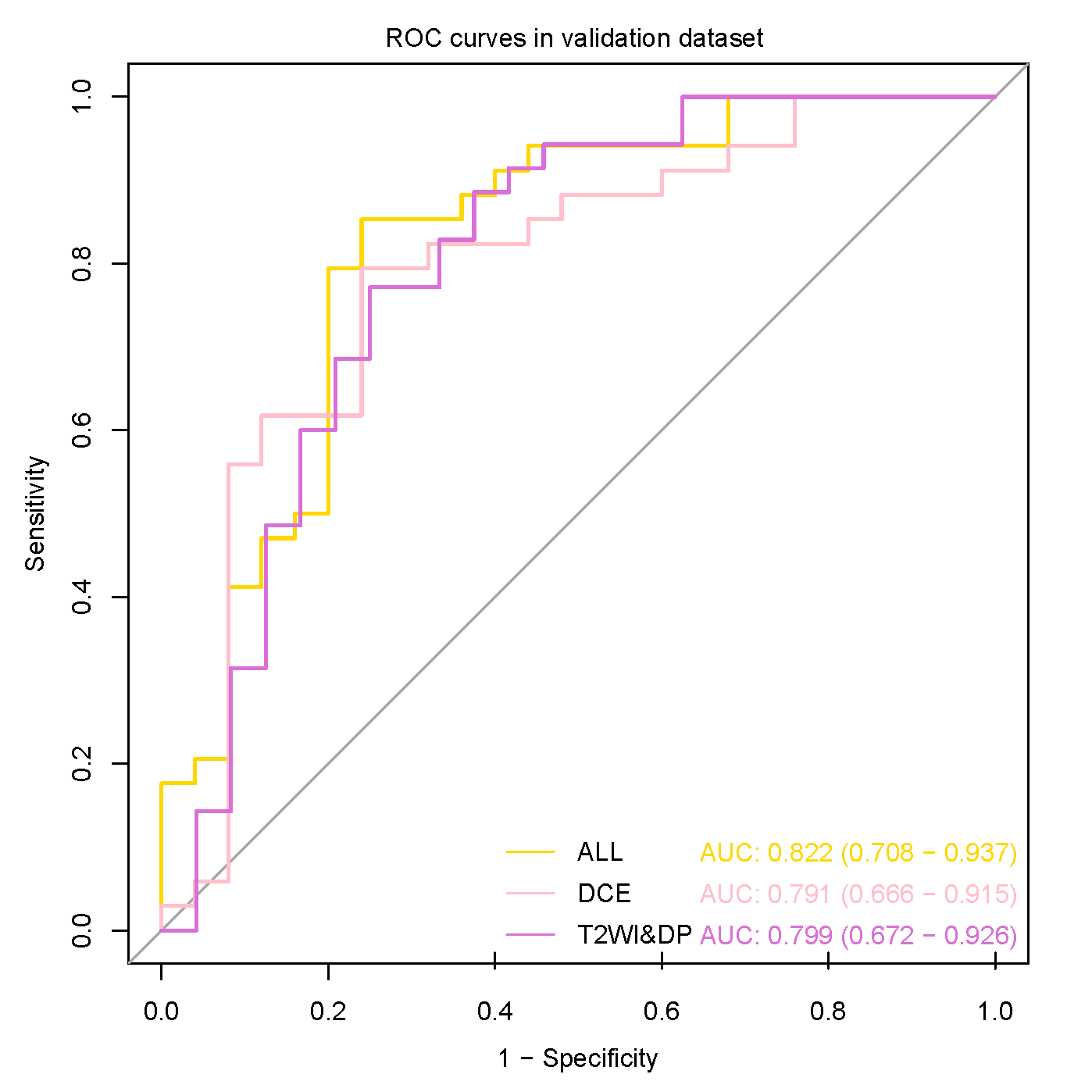

Supplement: Supplementary file 6 [file Image_5.tif]
