# Supplementary material for: Preoperative Prediction of Microvascular Invasion in Hepatocellular Carcinoma via Multi-Parametric MRI Radiomics
Source: Front Oncol. 2021 Mar 3;11:633596. doi: 10.3389/fonc.2021.633596 (PMC7968223; doi:10.3389/fonc.2021.633596)
Supplement: Supplementary file 7 [file Table_1.docx]

| **Supplementary Table S1.** Selected radiomics features for the multi-parametric MRI | | | | |
| --- | --- | --- | --- | --- |
| T_2_WI (n=11) | Intercept | 0.106 | — |  |
|  | Feature | -0.305 | MeanDeviation | Histogram |
|  |  | -0.579 | ClusterShade_AllDirection_offset4_SD | GLCM |
|  |  | 0.360 | ClusterShade_angle90_offset7 | GLCM |
|  |  | -0.442 | GLCMEnergy_AllDirection_offset7_SD | GLCM |
|  |  | 0.596 | LowIntensityLargeAreaEmphasis | GLSZM |
|  |  | -0.526 | ShortRunHighGreyLevelEmphasis_AllDirection_offset7_SD | RLM |
|  |  | -0.504 | ShortRunLowGreyLevelEmphasis_angle0_offset4 | RLM |
|  |  | -0.497 | HighGreyLevelRunEmphasis_AllDirection_offset1_SD | RLM |
|  |  | 0.456 | HighGreyLevelRunEmphasis_AllDirection_offset4_SD | RLM |
|  |  | -0.052 | LongRunHighGreyLevelEmphasis_AllDirection_offset7_SD | RLM |
|  |  | 0.305 | SphericalDisproportion | Formfactor |
| DWI (n=7) | Intercept | 0.089 | — |  |
|  | Feature | -0.316 | GLCMEnergy_AllDirection_offset4 | GLCM |
|  |  | -0.276 | GLCMEnergy_AllDirection_offset4_SD | GLCM |
|  |  | 0.125 | GLCMEnergy_angle45_offset1 | GLCM |
|  |  | -0.479 | LowIntensityEmphasis | GLSZM |
|  |  | 0.275 | ZonePercentage | GLSZM |
|  |  | -0.084 | HighGreyLevelRunEmphasis_AllDirection_offset1_SD | RLM |
|  |  | -0.465 | Compactness2 | Formfactor |
| ADC (n=6) | Intercept | -0.147 | — |  |
|  | Feature | 0.447 | Range | Histogram |
|  |  | 0.308 | ClusterShade_angle0_offset1 | GLCM |
|  |  | -5.768 | GLCMEnergy_angle0_offset7 | GLCM |
|  |  | 2.077 | GLCMEnergy_angle90_offset7 | GLCM |
|  |  | 0.313 | GLCMEntropy_AllDirection_offset7_SD | GLCM |
|  |  | -0.558 | Sphericity | Formfactor |
| AP (n=8) | Intercept | 0.253 | — |  |
|  | Feature | -0.333 | Percentile95 | Histogram |
|  |  | 0.043 | MinIntensity | Histogram |
|  |  | 0.271 | GLCMEntropy_angle135_offset7 | GLCM |
|  |  | 0.513 | Correlation_angle90_offset7 | GLCM |
|  |  | 0.125 | HaralickCorrelation_AllDirection_offset4_SD | GLCM |
|  |  | -0.645 | HighGreyLevelRunEmphasis_AllDirection_offset1_SD | RLM |
|  |  | -0.502 | ShortRunEmphasis_angle45_offset1 | RLM |
|  |  | 0.078 | HighGreyLevelRunEmphasis_AllDirection_offset4_SD | RLM |
| PP (n=6) | Intercept | -0.446 | — |  |
|  | Feature | -0.299 | MinIntensity | Histogram |
|  |  | 0.325 | kurtosis | Histogram |
|  |  | -4.452 | GLCMEnergy_angle135_offset7 | GLCM |
|  |  | 0.388 | InverseDifferenceMoment_AllDirection_offset7_SD | GLCM |
|  |  | -0.300 | HighGreyLevelRunEmphasis_AllDirection_offset7_SD | RLM |
|  |  | 0.148 | LongRunLowGreyLevelEmphasis_AllDirection_offset1_SD | RLM |
| DP (n=9) | Intercept | -0.171 | — |  |
|  | Feature | -0.428 | stdDeviation | Histogram |
|  |  | 0.334 | kurtosis | Histogram |
|  |  | 1.062 | ClusterProminence_angle90_offset7 | GLCM |
|  |  | 0.748 | ClusterShade_AllDirection_offset4 | GLCM |
|  |  | -1.556 | HaralickCorrelation_AllDirection_offset4_SD | GLCM |
|  |  | -0.320 | InverseDifferenceMoment_AllDirection_offset1_SD | GLCM |
|  |  | -0.421 | Correlation_AllDirection_offset4_SD | GLCM |
|  |  | -1.093 | SmallAreaEmphasis | GLSZM |
|  |  | -0.483 | LowIntensityLargeAreaEmphasis | GLSZM |
| Note. AP, arterial phase; PP, portal venous phase; DP, delay phase; GLCM, gray-level cooccurrence matrix; GLSZM, gray-level size zone matrix; RLM, run-length matrix. | | | | |
